# DIGIT-HF: Results From the Win Ratio Analyses

**DOI:** 10.1161/CIRCHEARTFAILURE.126.014396

**Published:** 2026-05-28

**Authors:** Xiaofei Liu, Nele Henrike Thomas, Dominik Berliner, Johannes Schwab, Andreas Rieth, Christina Strack, Sven Schallhorn, Samira Soltani, Lea Haebel, Welf Geller, Marija Zdravkovic, Martin Hülsmann, Heiko von der Leyen, Christian Veltmann, Stefan Störk, Michael Böhm, Johann Bauersachs, Armin Koch, Udo Bavendiek, Anika Großhennig, on behalf of the DIGIT-HF Study Group, Katharina Marx-Schütt

**Affiliations:** Uniklinik RWTH Aachen; Uniklinik RWTH Aachen; Kerckhoff-Klinik GmbH Bad Nauheim; Klinikum Westmünsterland Ahaus; Klinikum Westmünsterland Ahaus; CIMS Studienzentrum Bamberg GmbH Bamberg; CIMS Studienzentrum Bamberg GmbH Bamberg; Charité Campus Virchow Klinikum Berlin; Charité Campus Virchow Klinikum Berlin; Unfallkrankenhaus Berlin; Unfallkrankenhaus Berlin; Charité Universitätsmedizin Berlin-CBF Berlin; Charité Universitätsmedizin Berlin-CBF Berlin; Katholisches Klinikum Bochum gGmbH, St. Elisabeth-Hospital Bochum; Katholisches Klinikum Bochum gGmbH, St. Elisabeth-Hospital Bochum; Katholisches Klinikum Bochum gGmbH, Sankt Josef-Hospital Bochum; Katholisches Klinikum Bochum gGmbH, Sankt Josef-Hospital Bochum; Universitätsklinikum Bonn; Universitätsklinikum Bonn; Klinikum Links der Weser Herzzentrum Bremen; Klinikum Links der Weser Herzzentrum Bremen; Klinikum Coburg; Klinikum Coburg; Klinikum Lippe Detmold; Klinikum Lippe Detmold; Herzzentrum Dresden GmbH-Universitätsklinikum Dresden; Herzzentrum Dresden GmbH-Universitätsklinikum Dresden; Herzzentrum Dresden GmbH, Universitätsklinik an der Technischen Universität Dresden; Herzzentrum Dresden GmbH, Universitätsklinik an der Technischen Universität Dresden; Evangelisches Krankenhaus Düsseldorf; Evangelisches Krankenhaus Düsseldorf; Universitätsklinikum Düsseldorf; Universitätsklinikum Düsseldorf; Universitätsklinikum Essen; Universitätsklinikum Essen; Universitätsklinikum Freiburg; Universitätsklinikum Freiburg; Universitätsmedizin Göttingen; Universitätsmedizin Göttingen; Universitätsklinikum Halle; Universitätsklinikum Halle; Albertínen-Krankenhaus Hamburg; Albertínen-Krankenhaus Hamburg; Universitätsklinikum Hamburg-Eppendorf Hamburg; Universitätsklinikum Hamburg-Eppendorf Hamburg; Universitätsklinikum Heidelberg; Universitätsklinikum Heidelberg; Universitätsklinikum des Saarlandes Homburg/Saar; Universitätsklinikum Jena; Universitätsklinikum Jena; Herzzentrum Uniklinik Köln; Herzzentrum Uniklinik Köln; Leipzig Heart Institute GmbH Leipzig; Leipzig Heart Institute GmbH Leipzig; Universität Leipzig; Universität Leipzig; Universitätsklinikum Schleswig-Holstein Lübeck; Universitätsklinikum Schleswig-Holstein Lübeck; Otto-von-Guericke-Universität Magdeburg Universitätsklinikum Magdeburg A. ö. R. Magdeburg; Otto-von-Guericke-Universität Magdeburg Universitätsklinikum Magdeburg A. ö. R. Magdeburg; Universitätsmedizin Johannes-Gutenberg-Universität Mainz; Universitätsmedizin Johannes-Gutenberg-Universität Mainz; Universitätsklinikum Mannheim; Universitätsklinikum Mannheim; Universitätsklinikum Gießen und Marburg GmbH Standort Marburg; Universitätsklinikum Gießen und Marburg GmbH Standort Marburg; Internistische Praxis Dr. Taggeselle Markkleberg; Internistische Praxis Dr. Taggeselle Markkleberg; Carl von Basedow Klinikum Saalekreis GmbH Merseburg; Carl von Basedow Klinikum Saalekreis GmbH Merseburg; Kardiologisch-Angiologische Schwerpunktpraxis Mühldorf am Inn; Kardiologisch-Angiologische Schwerpunktpraxis Mühldorf am Inn; Universitätsklinikum München Campus Innenstadt München; Universitätsklinikum München Campus Innenstadt München; Klinikum Nürnberg Süd; St. Vinzenz-Krankenhaus GmbH Paderborn; St. Vinzenz-Krankenhaus GmbH Paderborn; Kardiologisches Zentrum Peine MVZ Peine; Kardiologisches Zentrum Peine MVZ Peine; Universitätsklinikum Regensburg; Universitätsklinikum Regensburg; Elblandklinikum Riesa; Elblandklinikum Riesa; MVZ Schwerin West GmbH Schwerin; MVZ Schwerin West GmbH Schwerin; Elbe Kliniken Stade-Buxtehude GmbH Stade; Elbe Kliniken Stade-Buxtehude GmbH Stade; Universitätsklinikum Würzburg; Medizinische Universität Wien; Medizinische Universität Wien; A. ö. Krankenhaus Sankt Josef Braunau GmbH Braunau; Institut za rehabilitaciju Belgrade; Institut Za Kardiovaskularne Bloesti “Dedinje” Belgrade; Klinicko Bolnicki Centar “Zvezdara”- Beograd Belgrade; Univerzitetski Klinicki Centar Nis Nis; Institut Za Lecenje I Rehabilitciju “Niska Banja” Nis Niska Banja; (Head of DMC), Charite Universitätsmedizin Berlin, Germany; (Statistician of DMC), Ruhr-Universität Bochum, Germany; (Medical Expert), HerzGefäßZentrum im Park Hirslanden, Switzerland; Medizinische Universität Innsbruck, Austria; Institut für Klinische Forschung Göttingen, Germany; Theresienkrankenhaus Mannheim, Germany; Universitätsmedizin Göttingen, Germany; (Sponsor Representative), Medizinische Hochschule Hannover, Germany; (Pharmacovigilance), Medizinische Hochschule Hannover, Germany; Institute for Biostatistics (X.L., N.H.T., A.K., A.G.), Hannover Medical School, Germany.; Department of Cardiology and Angiology (D.B., S. Schallhorn, S. Soltani, L.H., W.G., C.V., J.B., U.B.). Hannover Medical School, Germany.; Department of Cardiology, Paracelsus Medical University, Nuremberg, Germany (J.S.).; MVZ Kardiologie, Klinikum Neumarkt, Germany (J.S.).; Department of Cardiology, Kerckhoff-Klinik, Bad Nauheim, Germany (A.R.).; Department for Internal Medicine II, University Hospital Regensburg, Germany (C.S.).; Clinic for Internal Medicine, University Clinical Hospital Center Bezanijska Kosa, Faculty of Medicine, University of Belgrade, Serbia (M.Z.).; Universitätsklinik für Innere Medizin II, Abteilung Kardiologie, Medizinische Universität Wien, Austria (M.H.).; Orgenesis, Inc, Germantown (H.v.d.L.).; Hannover Medical School, Hannover, Germany (H.v.d.L.).; Center for Electrophysiology Bremen, Germany (C.V.).; Department Clinical Research and Epidemiology, Comprehensive Heart Failure Center Würzburg, and Department Internal Medicine I, University Hospital Würzburg, Germany (S. Störk).; Klinik für Innere Medizin III, HOMICAREM (HOMburg Institute for CArdioREnalMetabolic Medicine), Universitätsklinikum des Saarlandes, Saarland University, Germany (M.B.).

**Keywords:** benefit assessment, cardiac glycosides, clinical trial, digitoxin, heart failure, win ratio

DIGIT-HF (Digitoxin to Improve Outcomes in Patients With Advanced Chronic Heart Failure) was a randomized, double-blind, placebo-controlled, multicenter trial of digitoxin in heart failure with reduced ejection fraction.^1^ The trial met its primary objective, demonstrating superiority of digitoxin over placebo for time to first heart failure hospitalization (HFH) or all-cause death (whichever occurred first), with a favorable trend in all-cause mortality.^1^ To further support the robustness of these findings and capture treatment benefit across outcomes ordered by clinical relevance, we report results from win ratio analyses.

The hierarchical composite of time to all-cause death, number of HFHs, and time to first HFH was a prespecified secondary end point, assessed using the win ratio method.^1,2^ Each digitoxin-treated patient was compared with each placebo-treated patient. A comparison favored digitoxin if the digitoxin-treated patient survived longer, had fewer HFHs, or experienced a later first HFH than the corresponding placebo-treated patient; it favored placebo if the opposite was observed. If no difference was determined across the hierarchy, the pair was classified as a tie. To evaluate the impact of including a lower-priority functional outcome, change from baseline in New York Heart Association functional class (improvement, no change, or deterioration) was added post hoc as the lowest-priority outcome. Comparisons were made at the maximum common follow-up of each pair, which is more efficient than using a fixed time point. The trial was approved by the respective ethics committees, and all participants provided informed consent. Data are available upon reasonable request.

The Figure displays the proportions of wins for digitoxin and placebo and the remaining ties at each level of the hierarchy, together with win ratios up to each level (total wins with digitoxin divided by total wins with placebo), approximate 95% CIs, and *P* values.^2^ The proportions of ties resolved up to each level quantify the discriminatory capacity of the outcomes ordered according to clinical relevance.

**Figure.**
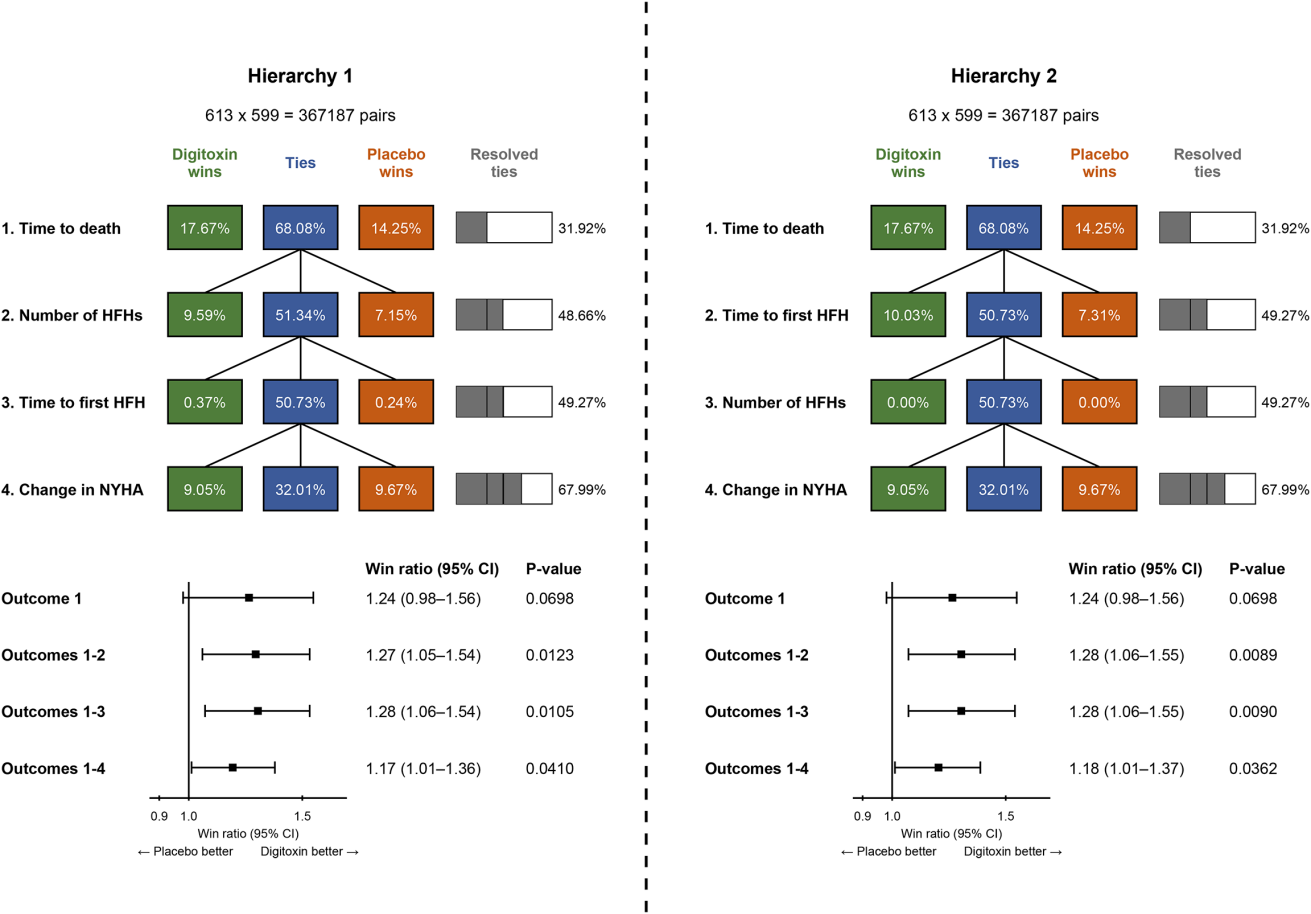
**Win ratios in the DIGIT-HF trial (Digitoxin to Improve Outcomes in Patients With Advanced Chronic Heart Failure).** Win ratios were derived from pairwise comparisons between 613 patients randomized to digitoxin and 599 patients to placebo (367 187 pairs). Hierarchy 1 consisted of time to all-cause death, number of heart failure hospitalizations (HFHs), time to first HFH, and change in New York Heart Association (NYHA) functional class. Hierarchy 2 reversed the order of the 2 HFH components. Proportions of wins for digitoxin and placebo and remaining ties at each level of the hierarchy as well as proportions of resolved ties up to each level are displayed. Win ratios up to each level with approximate 95% CIs and *P* values are presented.

Overall, 613 patients randomized to digitoxin and 599 patients to placebo generated 367 187 pairwise comparisons. When only time to death was considered, digitoxin won more often than placebo, yielding a win ratio of 1.24 (95% CI, 0.98–1.56; *P*=0.0698), indicating a survival benefit without statistical significance. This is consistent with the prespecified Cox model for time to death (hazard ratio, 0.86 [95% CI, 0.69–1.07]; *P*=0.1694).^1^

Adding HFH outcomes (number of HFHs and time to first HFH) provided additional discrimination between patients, resulting in more wins with digitoxin at these levels and nominally significant win ratios. This indicates an additional treatment benefit beyond mortality. The ordering of the 2 HFH components (hierarchy 1 versus hierarchy 2) had negligible impact. Adding the number of HFHs after time to first HFH had only minimal impact on tie resolution, suggesting that recurrent-event analyses are not inherently more informative than time-to-first-event analyses. These findings align with the primary DIGIT-HF results, in which similar effect estimates were observed for time to first HFH or death (hazard ratio, 0.82 [95% CI, 0.69–0.98]; *P*=0.0268) and for the total number of HFHs and death analyzed using a prespecified negative binomial model (rate ratio, 0.85 [95% CI, 0.67–1.09]; *P*=0.1993).^1^ Similar observations have been reported elsewhere.^3^

Whereas the primary end point of DIGIT-HF weighted HFH and death equally, the win ratio analysis prioritized death over first HFH, yielding a win ratio of 1.28 (95% CI, 1.06–1.55; *P*=0.0089). Consistent results were obtained when the win ratio was calculated directly for the primary end point as the sole outcome in the hierarchy (win ratio, 1.32 [95% CI, 1.09–1.60]; *P*=0.0043).

Adding New York Heart Association class further resolved ties. However, because digitoxin did not achieve more wins at this level, overall win ratios decreased, indicating no clear functional benefit beyond mortality and HFH. Nevertheless, this is more reassuring than the reverse scenario in which digitoxin achieved more wins only for New York Heart Association class.

DIGIT-HF was informative regarding the most clinically relevant outcomes, with ≈50% of pairwise comparisons resolved by death and HFH together and about 32% by mortality alone. The resolution pattern was similar to that observed in contemporary large-scale heart failure with reduced ejection fraction trials such as VICTORIA (Vericiguat Global Study in Subjects With Heart Failure With Reduced Ejection Fraction) and GALACTIC-HF (Global Approach to Lowering Adverse Cardiac Outcomes Through Improving Contractility in Heart Failure).^4,5^ This was likely attributable to the substantially longer follow-up in DIGIT-HF (median, 36 months), which allowed a sufficient number of events to accrue and thereby increased the trial’s discriminatory capacity. When trials evaluate comparable populations and similarly effective treatments, a higher proportion of ties resolved by death and HFH generally indicates greater robustness.

The win ratio does not quantify digitoxin treatment benefit on a directly interpretable clinical scale. Rather, it reflects the likelihood that digitoxin yields a better outcome than placebo across prespecified ordered dimensions. For this reason, the win ratio is less suitable as a primary efficacy measure in confirmatory trials. However, it provides a simple, model-free framework for a structured assessment of treatment benefit, in which the relative contribution of outcomes ordered by clinical relevance in determining the overall benefit is more informative than the magnitude or statistical significance of the estimate.

## Article Information

### Disclosures

Drs Bavendiek and Bauersachs are the study heads of the DIGIT-HF study and, together with Dr Koch, applied for the funding of DIGIT-HF described above. Drs Bavendiek, Bauersachs, Koch, von der Leyen, Veltmann, Störk, and Böhm are members of the DIGIT-HF trial Steering Committee. Dr Berliner was competitively selected for CORE100Pilot, which is an advanced clinician–scientist program cofunded by the Else Kröner Fresenius Foundation and the Ministry for Science and Culture of the State of Lower Saxony. Dr Berliner received honoraria for lectures/consulting from Abbott Vascular, AstraZeneca, Boehringer Ingelheim, Bristol Myers Squibb, Daiichi Sankyo, Edwards Lifesciences, and Pfizer, all unrelated to this article. Dr Strack reports reimbursement of travel expenses from Daiichi Sankyo, Sanofi, and Novartis, and honoraria for lectures from Pfizer and Organon, all unrelated to this article. Dr Hülsmann reports receiving research grants, speaker honoraria, and honoraria for advisory activities from Boehringer Ingelheim, AstraZeneca, Bayer, Roche, Diagnostics, Biopeutics, and Novartis, all unrelated to this article. Dr von der Leyen is a consultant and has received honoraria from Orgenesis, Inc. Dr Veltmann reports honoraria for lectures or consulting from Abbott, Medtronic, BMS, and Zoll, all unrelated to this article. Dr Störk reports receiving grant support from the Federal Ministry for Research and Education; speaker honoraria from AstraZeneca, Bayer, Boehringer, Novo Nordisk, Pfizer, Sanofi, and Servier, all unrelated to this article; and personal advisory roles with AstraZeneca, Bayer, Boehringer, Novartis, Novo Nordisk, Pfizer, Sanofi, and Servier, all unrelated to this article. He also holds unpaid leadership positions in national and European heart failure organizations. In addition, he received case fees for study conduct from Akcea, Alnylam, Amgen, AstraZeneca, Bayer, Boehringer, Cytokinetics, MSD, Novartis, Novo Nordisk, Pfizer, and Sanofi, all unrelated to this article. Dr Böhm is supported by the Deutsche Forschungsgemeinschaft (German Research Foundation; TTR 219, project number 322900939) and reports personal fees from Abbott, Amgen, AstraZeneca, Bayer, Boehringer Ingelheim, Cytokinetics, Edwards, Medtronic, Novartis, ReCor, Servier, and Vifor during the conduct of the study. Dr Bauersachs received honoraria for lectures/consulting from Novartis, Abbott, Bayer, Pfizer, Boehringer Ingelheim, AstraZeneca, Cardior, CVRx, BMS, Amgen, Edwards, Roche, and Zoll, all unrelated to this article; and research support for the department from Zoll, CVRx, Abiomed, Norgine, and Roche, all unrelated to this article. Dr Bavendiek received travel support and honoraria for lectures/consulting from Alnylam Pharmaceuticals, Amgen, AstraZeneca, Bayer Vital, Lilly, Novartis, and Pfizer, and institutional research support/funding from Alnylam Pharmaceuticals, all unrelated to this article. The other authors report no conflicts.

### Supplemental Material

DIGIT-HF Study Group

## Supplementary Material


