# Supplementary material for: DIGIT-HF: Results From the Win Ratio Analyses
Source: Circ Heart Fail. 2026 May 28;19(7):e014396. doi: 10.1161/CIRCHEARTFAILURE.126.014396 (PMC13390985; doi:10.1161/CIRCHEARTFAILURE.126.014396)
Supplement: Supplementary file 1 [file hhf-19-e014396-s001.pdf]

DIGIT-HF: results from the win ratio analyses

Supplemental Material

DIGIT-HF Study Group

Investigators by country

Germany
Katharina Marx-Schütt, Nikolaus Marx, Uniklinik RWTH Aachen
Andreas Rieth, Veselin Mitrovic, Kerckhoff-Klinik GmbH Bad Nauheim
Alessandro Cuneo, Sergiu Hicea, Klinikum Westmünsterland Ahaus
Andreas Ritze, Johannes Haas, CIMS Studienzentrum Bamberg GmbH Bamberg
Frank Edelmann, Tobias Trippel, Charité Campus Virchow Klinikum Berlin
Sebastian Winkler, Mirko Seidel, Unfallkrankenhaus Berlin
Bettina Heidecker, Arash Haghikia, Charité Universitätsmedizin Berlin-CBF Berlin
Monika Ernst, Christoph Hanefeld, Katholisches Klinikum Bochum gGmbH, St. Elisabeth-Hospital Bochum
Kristina Becker, Andreas Mügge, Katholisches Klinikum Bochum gGmbH, Sankt Josef-Hospital Bochum
Georg Nickenig, Can Öztürk, Universitätsklinikum Bonn
Rainer Hambrecht, Harm Wienbergen, Klinikum Links der Weser Herzzentrum Bremen
Johannes Brachmann, Steffen Schnupp, Klinikum Coburg
Ralph Oeckinghaus, Timo Aschenbrenner, Klinikum Lippe Detmold
Thomas Paul Gaspar, Stefan Ulbrich, Herzzentrum Dresden GmbH-Universitätsklinikum Dresden
Ruth Strasser, Stafanie Katzke, Herzzentrum Dresden GmbH, Universitätsklinik an der Technischen Universität Dresden
Christian Meyer, Bahram Wafaisade, Evangelisches Krankenhaus Düsseldorf
Daniel Scheiber, Malte Kelm, Universitätsklinikum Düsseldorf
Till Neumann, Hagen Kälsch, Universitätsklinikum Essen
Constantin von zu Mühlen, Sebastian Grundmann, Universitätsklinikum Freiburg
Kristian Hellenkamp, Tim Seidler, Universitätsmedizin Göttingen
Alexander Vogt, Jochen Dutzmann, Universitätsklinikum Halle
Herbert Nägele, Philipp Peitsmeyer, Albertínen-Krankenhaus Hamburg
Moritz Becher, Nina Fluschnik, Universitätsklinikum Hamburg-Eppendorf Hamburg
Udo Bavendiek, Johann Bauersachs, Medizinische Hochschule Hannover
Lutz Frankenstein, Tobias Täger, Universitätsklinikum Heidelberg
Michael Böhm, Ingrid Kindermann, Universitätsklinikum des Saarlandes Homburg/Saar
Christian Schulze, Julian Georg Westphal, Universitätsklinikum Jena
Roman Pfister, Stephan Rosenkranz, Herzzentrum Uniklinik Köln
Marcus Sandri, Holger Thiele, Leipzig Heart Institute GmbH Leipzig

Rolf Wachter, Michael Metze, Universität Leipzig
Tobias Graf, Christian Reil, Universitätsklinikum Schleswig-Holstein Lübeck
Rüdiger Braun-Dullaeus, Alexander Schmeißer, Otto-von-Guericke-Universität Magdeburg Universitätsklinikum Magdeburg A. ö. R. Magdeburg
Philip Wenzel, Tommaso Gori, Universitätsmedizin Johannes-Gutenberg-Universität Mainz
Michael Behnes, Ibrahim Akin, Universitätsklinikum Mannheim
Bernhard Schieffer, Wolfram Grimm, Universitätsklinikum Gießen und Marburg GmbH Standort Marburg
Jens Taggeselle, Antje Stumpp, Internistische Praxis Dr. Taggeselle Markkleberg
Roland Prondzinsky, Susanne Rode, Carl von Basedow Klinikum Saalekreis GmbH Merseburg
Norbert Schön, Brigitte Schön, Kardiologisch-Angiologische Schwerpunktpraxis Mühldorf am Inn
Stefan Kääb, Stefan Brunner, Universitätsklinikum München Campus Innenstadt München
Johannes Schwab, Matthias Pauschinger, Klinikum Nürnberg Süd
Andreas Götte, Sibylle Brandner, St. Vinzenz-Krankenhaus GmbH Paderborn
Uwe Gremmler, Birgit Gerecke, Kardiologisches Zentrum Peine MVZ Peine
Lars Maier, Bernhard Unsöld, Universitätsklinikum Regensburg
Markus Schwefer, Stefan Hettwer, Elblandklinikum Riesa
Stefan Rausch, Kyrill Rogacev, MVZ Schwerin West GmbH Schwerin
Sebastian Philipp, Torsten Lauf, Elbe Kliniken Stade-Buxtehude GmbH Stade
Stefan Störk, Caroline Morbach, Universitätsklinikum Würzburg

Austria
Martin Hülsmann, Suriya Prausmüller, Henrike Arfsten, Medizinische Universität Wien
Johann Auer, A. ö. Krankenhaus Sankt Josef Braunau GmbH Braunau

Serbia
Marija Zdravkovic, Klinicko bolnicki centar Bezanijska Kosa Belgrade
Dejan Spiroski, Institut za rehabilitaciju Belgrade
Dragana Kosevic, Institut Za Kardiovaskularne Bloesti "Dedinje" Belgrade
Natasa Markovic Nikolic, Klinicko Bolnicki Centar "Zvezdara"- Beograd Belgrade
Svetlana Apostolovic, Univerzitetski Klinicki Centar Nis Nis
Marina Deljanin Ilic, Institut Za Lecenje I Rehabilitciju “Niska Banja” Nis Niska Banja

Committees and Study Personnel

Trial Steering Committee (TSC)
Udo Bavendiek (Head of Study), Medizinische Hochschule Hannover, Germany
Johann Bauersachs (Head of Study), Medizinische Hochschule Hannover, Germany
Armin Koch (Statistician of TSC), Medizinische Hochschule Hannover, Germany
Christian Veltmann (Medical Expert), Elektrophysiologie Bremen, Germany
Michael Böhm (Medical Expert), Universitätsklinikum Homburg/Saar, Germany
Heiko von der Leyen (Medical Expert), Medizinische Hochschule Hannover, Germany
Stefan Störk (Medical Expert), Universitätsklinikum Würzburg, Germany

Data Monitoring Committee (DMC)
Stefan Anker (Head of DMC), Charite Universitätsmedizin Berlin, Germany
Hans J. Trampisch (Statistician of DMC), Ruhr-Universität Bochum, Germany
Paul Mohacsi (Medical Expert), HerzGefäßZentrum im Park Hirslanden, Switzerland
Gerhard Pölzl, Medizinische Universität Innsbruck, Austria

Clinical Event Adjudication Committee (CEAC)
Ulrich Tebbe, Institut für Klinische Forschung Göttingen, Germany
Markus Haas, Theresienkrankenhaus Mannheim, Germany
Stephan von Haehling, Universitätsmedizin Göttingen, Germany

National Lead Investigators
Udo Bavendiek (Head of study), Medizinische Hochschule Hannover, Germany
Marija Zdravkovic, Klinicko bolnicki centar Bezanijska Kosa Belgrade, Serbia
Martin Hülsmann, Medizinische Universität Wien, Austria

Principal Investigators and Sponsor
Udo Bavendiek (Head of Study), Medizinische Hochschule Hannover, Germany
Johann Bauersachs (Head of Study), Medizinische Hochschule Hannover, Germany
Armin Koch (Statistician of TSC), Medizinische Hochschule Hannover, Germany
Christoph Schindler (Sponsor Representative), Medizinische Hochschule Hannover, Germany
Dirk O. Stichtenoth (Pharmacovigilance), Medizinische Hochschule Hannover, Germany
